# Use of functional gene arrays for elucidating in situ biodegradation

**DOI:** 10.3389/fmicb.2012.00339

**Published:** 2012-09-21

**Authors:** Joy D. Van Nostrand, Zhili He, Jizhong Zhou

**Affiliations:** ^1^Institute for Environmental Genomics, University of OklahomaNorman, OK, USA; ^2^Department of Microbiology and Plant Biology, University of OklahomaNorman, OK, USA

**Keywords:** functional gene microarray, GeoChip, microbial communities, bioremediation, biodegradation

## Abstract

Microarrays have revolutionized the study of microbiology by providing a high-throughput method for examining thousands of genes with a single test and overcome the limitations of many culture-independent approaches. Functional gene arrays (FGA) probe a wide range of genes involved in a variety of functions of interest to microbial ecology (e.g., carbon degradation, N fixation, metal resistance) from many different microorganisms, cultured and uncultured. The most comprehensive FGA to date is the GeoChip array, which targets tens of thousands of genes involved in the geochemical cycling of carbon, nitrogen, phosphorus, and sulfur, metal resistance and reduction, energy processing, antibiotic resistance and contaminant degradation as well as phylogenetic information (*gyrB*). Since the development of GeoChips, many studies have been performed using this FGA and have shown it to be a powerful tool for rapid, sensitive, and specific examination of microbial communities in a high-throughput manner. As such, the GeoChip is well-suited for linking geochemical processes with microbial community function and structure. This technology has been used successfully to examine microbial communities before, during, and after *in situ* bioremediation at a variety of contaminated sites. These studies have expanded our understanding of biodegradation and bioremediation processes and the associated microorganisms and environmental conditions responsible. This review provides an overview of FGA development with a focus on the GeoChip and highlights specific GeoChip studies involving *in situ* bioremediation.

## INTRODUCTION

As the most phylogenetically and functionally diverse group of organisms on the planet (estimated 2000–50,000 microbial species per gram of soil; Torsvik et al., 1990; Hong et al., 2006; Schloss and Handelsman, 2006; Roesch et al., 2007), microorganisms are critical to ecosystem functioning and are involved in the biogeochemical cycling of carbon, nitrogen, sulfur, phosphorus, and metals, as well as degradation or stabilization of contaminants in the environment. However, because a vast majority (>99%) of microorganisms remain uncultured (Amann et al., 1995; Fuhrman and Campbell, 1998; Whitman et al., 1998), culture-independent approaches must be used to gain a comprehensive picture of microbial communities. However, many of the culture-independent methods, such as 16S rRNA gene-based cloning or quantitative PCR, require a PCR amplification step, which introduces well-known biases (Suzuki and Giovannoni, 1996; Warnecke et al., 1997; Lueders and Friedrich, 2003). In addition, since many functional genes have too much variance or too few sequences available, conserved PCR primers cannot be designed for many functional genes. Even if primers could be designed for many functional genes, performing PCR with many different primer sets would be cost- and time-prohibitive.

Microarrays allow the examination of thousands of genes at one time without the need for PCR amplification of each gene. Since microarrays were first shown to be valuable for the study of microbial communities (Guschin et al., 1997), several types have been designed to examine microbial communities. These include (i) phylogenetic oligonucleotide arrays (POA), designed to examine phylogenetic relatedness or community composition using 16S rRNA or other conserved phylogenetic genes (Small et al., 2001; Loy et al., 2002; Wilson et al., 2002; Brodie et al., 2006); (ii) community genome arrays (CGA), designed to examine the relatedness of microbial species or strains or to identify community members using whole-genomic DNA probes (Wu et al., 2004; Zhang et al., 2004); (iii) metagenomic arrays (MGA), designed as a high-throughput screening method using environmental clone library inserts as probes (Sebat et al., 2003; Mockler and Ecker, 2005; Gresham et al., 2008); (iv) whole-genome ORF arrays (WGA), designed to examine gene expression of individual microorganisms using probes for all ORFs in one or more genomes (Wilson et al., 1999), but can also be used for comparative genomics (Murray et al., 2001); and (v) functional gene arrays (FGAs), designed to examine multiple functional genes at one time using probes for key genes involved in microbial functional processes of interest (Wu et al., 2001; He et al., 2007, 2010a). This review will focus on FGAs.

### MICROARRAYS VS. HIGH-THROUGHPUT SEQUENCING

While this review focuses on functional gene microarrays, high-throughput sequencing has become an increasingly popular choice for examining and monitoring microbial communities and is even being used for metatranscriptome analysis (van Vliet, 2010). However, while many of the technical challenges of microarrays and high-throughput sequencing have been overcome, each still has some distinct advantages and disadvantages, which make them ideal as complementary approaches: (i) Random sampling errors. In most sequencing studies, only a small proportion of the microbial community is actually sampled (McKenna et al., 2008) and while theoretically with true random sampling the probability of sampling the same fraction of the community multiple times is low (Zhou et al., 2008), one would expect that dominant populations would have a greater chance of being sampled multiple times. These sampling errors can result in low reproducibility between technical replicates (17.2 ± 2.3% for two replicates; 8.2 ± 2.3% for three; Zhou et al., 2011). Microarrays, in contrast, interrogate all samples against the same set of sequences (probes), so that the same population is sampled each time. (ii) Relative abundance. Abundance of individual species will vary greatly within microbial communities. With sequencing-based approaches there will be a bias toward the most abundant sequences in the environment so that many of the obtained sequences will represent the most abundant species/sequences while possibly missing lesser abundant species/sequences. Microarrays are not affected in the same way since lesser abundance sequences will still hybridize to their corresponding probe and as long as it is above the detection limit, it will be detected. (iii) New sequence detection. One of the greatest advantages of sequencing is that new sequences are easily detected since any sequences in the sample can be sequenced (open system). Microarrays, in contrast, can detect only the limited number of sequences covered by the probe set on the array (closed system), as such, it is not able to detect new sequences. Some new microarray techniques have been developed to allow the discovery of new sequences. Capture microarrays have been developed, which use lower stringency conditions to hybridize or “capture” sequence variants (Albert et al., 2007; Okou et al., 2007). These captured sequences are then washed off and sequenced. An array with probes specific for viral families has been developed that uses the hybridization pattern to classify novel viruses (Wang et al., 2002; Ksiazek et al., 2003). As such, microarray and sequencing approaches could be used to maximize the benefits and minimize the deficiencies of each.

### DEVELOPMENT OF FUNCTIONAL GENE ARRAYS

The first FGA developed used PCR-amplicon probes and targeted four N-cycling genes (*nirS*, *nirK*, *amoA*, and *pmoA*; Wu et al., 2001). However, since PCR-amplicons were used, only a limited number of genes could be included because conserved primers can only be designed for a few functional genes. In addition, it would be cost- and time-prohibitive to amplify genes from hundreds of microorganisms or clones in order to achieve a truly diverse probe set. Most microarrays now use oligonucleotide probes, which are more specific (Zhou, 2003), can be easily customized (Denef et al., 2003; Zhou, 2003; Gentry et al., 2006), and are relatively inexpensive.

Since then several different FGAs have been developed, although most of these cover only a limited number of genes and focus on specific functional groups or locations. For example, FGAs have been designed to examine methanotrophs (Bodrossy et al., 2003, 2006; Stralis-Pavese et al., 2004), N-cycling genes (Taroncher-Oldenburg et al., 2003; Jenkins et al., 2004; Steward et al., 2004; Zhang et al., 2007a), pathogens and virulence factors (Call et al., 2003; Kostić et al., 2005; Cleven et al., 2006; Miller et al., 2008; Palka-Santini et al., 2009), rhizobial isolates (Bontemps et al., 2005), and acid mine drainage (AMD) and bioleaching systems (Yin et al., 2007).

The most comprehensive FGAs reported to date are the GeoChip arrays. The GeoChip 1.0 had 2006 oligonucleotide probes (50-mers) for genes involved in nitrification, denitrification, nitrogen fixation, methane oxidation, sulfate reduction (Tiquia et al., 2004), organic contaminant degradation, and metal resistance (Rhee et al., 2004). This array was used in several studies examining microbial communities at uranium (U)-contaminated sites (Wu et al., 2006a; Waldron et al., 2009), in the Gulf of Mexico (Wu et al., 2008), and under different land use strategies (Zhang et al., 2007b) and showed FGAs to be useful for microbial community studies. GeoChip 2.0 was developed to provide a truly comprehensive probe set for multiple functional gene categories and to provide increased specificity for highly homologous gene variants (He et al., 2007). GeoChip 2.0 contains 24,243 (50-mer) oligonucleotide probes targeting ~10,000 functional genes from 150 gene families involved in the geochemical cycling of C, N, and P cycling, sulfate reduction, metal reduction and resistance, and organic contaminant degradation. This array has been used in numerous studies to examine microbial communities at metals contaminated sites (Gao et al., 2007; Van Nostrand et al., 2009, 2011), oil or diesel-contaminated sites (Rodríguez-Martínez et al., 2006; Liang et al., 2009a,b), coral mucus (Kimes et al., 2009), lake or river samples (Taş et al., 2009; Parnell et al., 2010), deep sea samples (Mason et al., 2009; Wang et al., 2009), Antarctic soils (Yergeau et al., 2007) and to examine the taxa–area relationship (Zhou et al., 2008).

GeoChip 3.0 covers 56,990 sequences from 292 gene families, greatly increasing the number of genes and categories covered compared to GeoChip 2.0 and added new control features (He et al., 2010a). New gene categories include antibiotic resistance, energy processing, and phylogenetic markers (i.e., *gyrB*). A set of 16S rRNA gene probes were added as positive controls, human, plant, or hyperthermophile gene probes were added as negative controls, and a common oligo reference standard (CORS) was added for data normalization and comparison. The CORS is composed of an artificial sequence probe that is co-spotted with each gene probe and the complementary CORS target, labeled with a contrasting fluorescent dye to the sample, which is then spiked into each sample prior to hybridization (Liang et al., 2010). The signal intensity of the CORS probe can then used to normalize the signal intensity of the sample and allows comparison of samples hybridized at different times. In addition, a computational pipeline has been developed for GeoChip probe design and data analysis. The GeoChip 3.0 has been used to examine microbial communities associated with elevated CO_2_ (He et al., 2010b), to examine communities within coal formation production waters (Wawrik et al., 2012b) or rhizosphere communities in As-contaminated sites (Xiong et al., 2010).

GeoChip 4.0, the newest version, is synthesized by Nimblegen (Madison, WI, USA) in their 12-plex format and contains 83,992 probes targeting 152,414 genes in 410 gene categories (Lu et al., 2012a). In addition to added genes in most categories, new categories added include stress response, antibiotic resistance, and bacteriophage genes. It has been used to examine microbial communities during the 2010 Gulf oil spill (Lu et al., 2012a),

## GEOCHIP DESIGN

### PROBE DESIGN AND SELECTION

GeoChip covers a wide range of functional genes and currently includes sequences from bacteria, Achaea, fungi, and viruses. The first step in designing new probes for the array is deciding which processes should be included. Then genes for enzymes or proteins that are key to the process of interest are selected. These could be catalytic subunits or proteins with recognition sites or that provide functional specificity. Next, keywords are selected to search public sequence databases (e.g., GenBank). The keywords should be as broad as possible since proteins from different microorganisms may be annotated differently or have more general or specific annotations. Once the sequences are downloaded, they are confirmed by HMMER alignment^1^with preselected seed sequences. The seed sequences are those sequences for which the protein identity and function have been experimentally confirmed. This is a critical step in the design process and these sequences should be selected with care. The HMMER confirmed sequences are then used to design gene- or group-specific 50-mer oligonucleotide probes using new versions of the CommOligo software (Li et al., 2005) and experimentally determined criteria based on sequence homology (≤90% identity for gene-specific probes, and ≥96% for group-specific probes), continuous stretch length (≤20 bases for gene-specific probes, and ≥35 for group-specific probes), and free energy (≥35 kJ mol^-^^1^ for gene-specific probes, and ≤60 kJ mol^-^^1^ for group-specific probes; He et al., 2005b; Liebich et al., 2006). The probes are then BLASTed against the GenBank database to confirm specificity. Keywords, downloaded sequences, seed sequences, HMMER confirmed sequences, and designed probes are stored in corresponding databases for use in future array updates.

The newly designed probe sets can then be commercially synthesized. Several options are available for producing arrays. Synthesized oligonucleotide probes can be spotted onto nylon membranes (Steward et al., 2004) or glass slides (Taroncher-Oldenburg et al., 2003; Rhee et al., 2004; Tiquia et al., 2004). Glass slides are more frequently used since they have less background fluorescence (Schena et al., 1995, 1996) and allow higher probe density (Ehrenreich, 2006). Probes can also be added to slides using bubble Jet printing (Okamoto et al., 2000), laser-induced forward transfer (Serra et al., 2004), or photolithography (Chen et al., 2009). In addition, a few companies, such as Agilent or Affymetrix, synthesize custom microarrays using a customer’s probe set.

### TARGET PREPARATION

GeoChip can be hybridized with either DNA or RNA. Most DNA samples used for GeoChip analysis are extracted using a well-established freeze-grind method with detergent lysis (Zhou et al., 1996; Hurt et al., 2001) since it provides high molecular weight DNA, important for later amplification steps. The use of RNA presents some challenges as mRNA is unstable and has a low abundance in environmental samples. Several papers have described methods for extracting environmental RNA, including a protocol for the dual extraction of both DNA and RNA (Hurt et al., 2001; Burgmann et al., 2003) or RNA alone (McGrath et al., 2008; Poretsky et al., 2009a). Methods for mRNA enrichment include size separation by gel electrophoresis (McGrath et al., 2008) or use of commercial kits [MICROBExpress (Ambion) and/or mRNA-ONLY (Epicentre Biotechnologies); Poretsky et al., 2009b; Mettel et al., 2010]. Size separation obtained 115–155 ng mRNA from 4.6–5.3 μg total RNA (McGrath et al., 2008). Using commercial kits, Mettel et al. (2010) were able to obtain 140–530 ng of mRNA from 0.4–2.0 μg total RNA per 0.5 g soil.

Nucleic acid quality is of great importance for microarray analysis. DNA and RNA should have an A_260_ to A_280_ ratio ~1.8 and >1.9, respectively and an A_260_ to A_320_ ≥ 1.7. The A_260_ to A_320_ ratio is most important in determining microarray success (Ning et al., 2009). Some environmental samples, especially those with high humics, can be difficult to purify up to the necessary level. A gel purification strategy followed by a phenol–chloroform–butanol extraction (Xie et al., 2007; Liang et al., 2011) has been successful with a wide range of soil and sediment samples.

Large amounts of DNA (e.g., 1 μg) or RNA (e.g., 5 μg) are needed for GeoChip hybridization. However, it can be difficult to get sufficient quantities of nucleic acid from some types of samples (e.g., water) or the sample is too difficult or impossible to replace to use such large quantities of nucleic acid. In this case, amplification of DNA or RNA can be done using either whole community genome amplification (WCGA; Wu et al., 2006a) or whole community RNA amplification (WCRA; Gao et al., 2007). WCGA uses the Templiphi 500 amplification kit (phi 29 DNA polymerase, GE Healthcare, Piscataway, NJ, USA) with a modified amplification buffer and using 1–100 ng DNA provides a sensitive (10 fg detection limit) and representative amplification (<0.5% of amplified genes showed >2-fold difference from unamplified; Wu et al., 2006a). WCRA provides a representative amplification with 50–100 ng of starting material.

There are commercial kits available for microbial RNA amplification such as the MessageAmp^TM^ II-Bacteria RNA Amplification Kit (Life Technologies, Grand Island, NY, USA). There are also other commercially available methods for WCGA. Wang et al. (2011) compared two of these (*Bacillus stearothermophilus* DNA polymerase (*Bst*) and REPLI-g; Qiagen, Valencia, CA, USA) with the modified Templiphi kit (Wu et al., 2006a). The amplification bias for all methods was relatively low (<3-fold). Less bias was observed with REPLI-g and Templiphi for pure culture DNA and with REPLI-g for community DNA while *Bst* showed the least inhibition by lesser quality DNA.

The amplified (or unamplified) nucleic acids are directly labeled with a fluorescent dye (Cy3 or Cy5) using random priming with the Klenow fragment of DNA polymerase for DNA (Wu et al., 2006a) or Superscript^TM^ II/III RNase H-reverse transcriptase for RNA (He et al., 2005b). The labeled DNA/RNA is then purified and dried for hybridization.

### HYBRIDIZATION AND IMAGE ANALYSIS

The labeled nucleic acids are then hybridized to the microarray at 42–50°C with 40–50% formamide (He et al., 2007, 2010a,b; Lu et al., 2012a). Hybridization specificity can be adjusted by varying the temperature or the formamide concentration (the effective hybridization temperature increases by 0.6°C for every 1% of formamide). Hybridized slides are then scanned and analyzed by quantifying the pixel density (intensity) of each spot using image analysis software. Commercial manufacturers often have their own analysis software or other microarray analysis software can be used, such as GenePix Pro (Molecular Devices, Sunnyvale, CA, USA), GeneSpotter (MicroDiscovery, San Diego, CA, USA), or ImaGene (BioDiscovery, El Segundo, CA, USA). For GeoChip data, there is a data analysis pipeline^2^ for rapid preprocessing and data analysis. Poor and low quality spots and outliers, based on Grubbs’ test of outliers (Grubbs, 1969), are removed and then the signal intensities of all spots are normalized. Positive spots can be determined using signal-to-noise ratio [SNR = (signal mean-background mean)/background standard deviation], signal-to-both-standard-deviations ratio [SSDR = (signal mean-background mean)/(signal standard deviation-background standard deviation)] (He and Zhou, 2008), or signal-to-background ratio (SBR = signal mean/background mean) (Loy et al., 2002).

### DATA ANALYSIS

Due to the large volume of data obtained from GeoChip, data analysis can be very challenging. The data has a multivariate structure and the number of variables is much larger than the number of observations (*p*≫*n*). To assist users with data analysis steps, a pipeline is available which performs many of the common analyses^3^. Some common descriptive statistics used include relative abundance of gene categories or subcategories, richness and diversity (α and β) indices, and percentages of gene overlap between samples. To compare the overall community structure, unconstrained ordination [principal component analysis (PCA) and correspondence analysis (CA)] to reduce the dimensionality of variables in order to maximize the visible variability of the data or hierarchical cluster analysis (HCA), which groups communities based on the similarity of their gene profiles, can be used. To compare communities, response ratios, which compare the signal intensity of genes between conditions (Luo et al., 2006; Liang et al., 2009a), *t*-tests, ANOVA, and dissimilarity tests can be used. Several methods can be used to examine the relationship between communities and environmental parameters. These include constrained ordination, such as canonical correspondence analysis (CCA; ter Braak, 1986), distance-based redundancy analysis (db-RDA; Legendre and Anderson, 1999), variation partitioning analysis (VPA; Økland and Eilertsen,1994; Ramette and Tiedje, 2007), and Mantel test. A relatively new analysis method is the random matrix theory-based (Mehta, 1990) neural network analysis (NNA) used to examine gene relationships within microbial ecological networks (Zhou et al., 2010).

## IMPORTANT CHALLENGES FOR MICROARRAY ANALYSIS

### NUCLEIC ACID QUALITY

Having high-quality nucleic acids (non-degraded, large fragments to improve amplification yields, absence of inhibitors or contaminants which may impede subsequent amplification and labeling steps) is the most important criterion for successful microarray experiments. Nucleic acids can be purified using commercial kits although the presence of humic acids and other contaminants can still be a problem. If large amounts of DNA are present, an agarose gel purification followed by phenol–chloroform–butanol extraction (Xie et al., 2007; Liang et al., 2009b) can be used, but large amounts of DNA are lost with this method so it is not practical for low abundance samples. So, better purification methods with high recovery yields are needed.

### SEQUENCE COVERAGE

One of the main objectives in developing FGAs was to provide a truly comprehensive probe set (He et al., 2007). Each new GeoChip version has expanded the coverage of gene variants and expands the number of genes covered (He et al., 2007, 2010a; Lu et al., 2012a). This continued expansion is challenging as the number of gene sequences available is constantly increasing as new sequences are being submitted to public databases. While the GeoChip design pipeline^2^ has an automated update feature which uses the previously selected key words and seed sequences to search the NCBI database, downloading new sequences and designing probes is still time consuming due to the sheer volume of sequences available. As such, better and faster computation systems are needed. In addition, available microarray probe density limits are rapidly being approached as the number of GeoChip probes increases. So, new methods of array construction to increase probe density are needed.

### SPECIFICITY AND SENSITIVITY

Two key issues for microarray hybridization of microbial communities are specificity and sensitivity since environmental communities can have such vast diversities. Both of these conditions can be improved at various stages of microarray design, construction, target preparation, or hybridization. During probe design, determining the best criteria for probe design, such as using experimentally determined design criteria (He et al., 2005b; Liebich et al., 2006) can improve specificity, thus decreasing the number of false positives (He et al., 2007). Probe length also affects specificity and sensitivity; longer probes are more sensitive, but less specific (Denef et al., 2003; He et al., 2005a).

The method of array synthesis can also affect sensitivity and specificity. Increasing the probe concentration per spot can increase sensitivity (Cho and Tiedje, 2002; Relógio et al., 2002; Zhou and Thompson, 2002). However, this may also decrease specificity by decreasing the overall probe signal intensity (Denef et al., 2003). The choice of array surface can also be important as use of unmodified array slides can decrease background fluorescence thus requiring a lower signal fluorescence for detection (Kumar et al., 2000; Gudnason et al., 2008).

Target preparation strategies can also affect these parameters. Amplification of community DNA can increase sensitivity. WCGA was able to representatively amplify 1–250 ng of community DNA (Wu et al., 2006a), increasing the detection limit from 25 ng to 10 pg (2 bacterial cells); however, using such small quantities of starting material greatly increases the amplification bias compared to the bias observed with 1 ng of DNA. Labeling methods can also affect sensitivity. For example, cyanine dye-doped nanoparticles or tyramide signal amplification labeling are able to increase sensitivity 10-fold (Denef et al., 2003; Zhou and Zhou, 2004).

Hybridization conditions can also be used to increase specificity and sensitivity. Temperature and formamide concentration can be modified to adjust stringency thus altering specificity (Wu et al., 2001). A lower hybridization solution volume (Shalon et al., 1996) and mixing during hybridization (Adey et al., 2002) have both been shown to increase sensitivity. Decreasing ozone levels, which can degrade cy-dye signal (Branham et al., 2007), can also improve sensitivity.

### MONITORING GENE ACTIVITY

Most GeoChip analysis has involved the use of DNA, so that only gene abundance can be determined. These changes can be used to infer microbial activity, but cannot provide direct proof of activity. mRNA can be used for FGA analysis to monitor activity (Dennis et al., 2003; Bodrossy et al., 2006; Gao et al., 2007; Wawrik et al., 2012a), although as mentioned above, working with environmental RNA can be challenging. Stable isotope probing (SIP) has also been used with GeoChip to monitor microbial activity (Leigh et al., 2007).

Gao et al. (2007) used amplified community mRNA from a denitrifying fluidized bed reactor to examine microbial activity. Genes for nitrate and nitrite reduction, organic contaminant degradation, sulfite reduction, and polyphosphate kinase were detected, consistent with reactor operation (Gao et al., 2007). Another study used amplified community mRNA to examine nitrate utilization in marine bacterial communities (Wawrik et al., 2012a). Hybridization results indicated activity by *ureC*, *nirS*, *nirK*, *narG*, *nosZ*, *napA*, *nrfA*, *amoA*, and *nifH* genes, indicating that urea cycling, denitrification, dissimilatory nitrate, nitrite reduction, and N fixation were occurring (Wawrik et al., 2012a).

Another method of monitoring microbial activity with GeoChip is to combine it with SIP (Leigh et al., 2007). Microcosms were set up from soil samples collected from the root zone of a tree growing in a PCB-contaminated site and fed ^13^C-labeled or unlabeled biphenyl. Genes involved in biphenyl degradation were detected as were other organic contaminant degradation genes including those for degradation of benzoate, catechol, naphthalene, and phenol.

## APPLICATION OF GEOCHIP TO BIOREMEDIATION STUDIES

### METALS CONTAMINATED SITES

Several GeoChip-related studies have examined microbial communities from U-contaminated groundwater at the U.S. Department of Energy (DOE) Oak Ridge Integrated Field Research Challenge (OR-IFRC) site. Groundwater samples covering a range of contamination levels and an uncontaminated background sample were compared using GeoChip 1.0 (Wu et al., 2006a). Samples from the uncontaminated site and those with lower levels of contaminants had higher functional gene diversity and gene numbers. In addition, as expected based on the contaminants present at this site, genes for denitrification, organic contaminant degradation, metal resistance, and sulfite reduction (*dsr*) were detected. A similar sample set using the same array was examined in greater detail in a later study (Waldron et al., 2009). In this study, sulfate, pH, U, and Tc were found to be the most important drivers in determining the microbial community structure, with pH and the combination of U and Tc explaining ~21% of the variance observed or 29–40% when all four variables were included.

Another study at this site examined a pilot-scale field bioremediation system which used ethanol as an electron donor to stimulate microbial communities and immobilize U(VI) by reduction to U(IV) (Luo et al., 2006; Wu et al., 2006b,c). GeoChip 2.0 was used to examine the microbial communities during different phases of operation. A period of active U(VI) reduction occurred after initial start-up (days 137–304). During this period U(VI) reduction was relatively rapid and genes associated with denitrification, sulfate reduction, and Fe(III) reduction increased in abundance, suggesting that these populations were involved in U(VI) reduction (Van Nostrand et al., 2011). This active reduction was followed by a maintenance period during which the low level of U(VI) was maintained, and the denitrifying, sulfate- and Fe(III)-reducing communities remained in higher abundance. Next, the stability of the bioreduced U(IV) was examined by allowing the system to enter periods of starvation (ethanol injections were halted) and reoxidation (dissolved O_2_ entered the system). The functional communities showed distinct clustering patterns based on whether the system received ethanol or not, indicating a shift in community structure with the return of ethanol injections (Van Nostrand et al., 2009). While total gene numbers increased once ethanol injection was restarted, the relative abundance of each gene group changed little during and after starvation, indicating a functionally diverse community which could be stimulated after adverse conditions. Chemical oxygen demand (COD, i.e., ethanol) was the most important driver in determining community structure, but temperature, sulfate, and U(VI) were also important.

In this same remediation system, the sediment microbial community was examined with GeoChip 2.0 after 2 years of operation (Xu et al., 2010). Sediment samples were collected from 11 wells, 5 from the outer loop and 6 from the inner loop. Results revealed significant differences between the microbial communities in the inner and outer loops. The inner loop communities had higher gene numbers and greater diversity than those in the outer loop and inner and outer loop samples were grouped separately based on hierarchical clustering and principle component analysis, indicating that the ethanol injections stimulated the microbial communities in the inner loop. In addition, genes important for U(VI) reduction such as cytochrome *c*, *dsr*, and denitrification as well as genes involved in metal resistance and organic contaminant degradation were enriched in the inner loop where electron donor was added. This study demonstrated the importance of U(VI)-reducing populations for the maintenance of reducing U(IV) in this bioremediation system.

Another GeoChip 2.0 study examined groundwater microbial communities at a field site examining the use of acetate to stimulate U(VI)-reducing microorganisms in the subsurface at the Old Rifle site, a former U ore processing facility in Rifle CO (Liang et al., 2012). The study compared communities taken during a shift from sulfate to Fe(III)-reducing conditions. The overall community structure changed with the switch from Fe(III)- to sulfate-reducing conditions and were reflective of the redox conditions at the site. Sulfate-reducing and methane-generating microorganisms increased in abundance under sulfate-reducing conditions. Acetate, U(VI) and redox potential were important environmental variables in determining the microbial community structure.

Xie et al. (2011) examined five AMD sites in China using GeoChip 2.0 to determine the functional diversity and metabolic potential of microbial communities in these sites and to determine how the communities responded to environmental conditions. The sites showed a great deal of variability in regards to the microbial communities with ~150–1000 functional genes detected in each sample. Most of the genes represented on the GeoChip that were involved in C, N, S cycling and metal resistance were detected in all of the AMD sites. Results indicated that the immediate environmental conditions were important in forming the variations in the functional structure of microbial communities as opposed to spatial distance. There was a positive correlation between Zn resistance gene abundance and Zn concentration but not for other metals. However, the concentrations of B, Co, Cu, La, Mg, and S were significantly correlated with the community structure in these communities. Overall, results suggested that AMD microbial communities may not be as simple as previously thought.

GeoChip 2.0 has also been used to probe pure culture isolates for the presence of specific genes. Four Ni-resistant Gram-positive actinomycetes were hybridized to GeoChip to get a better idea of what metal resistance genes were present (Van Nostrand et al., 2007). Genes associated with resistance to Al, As, Cd, Cr, Cu, Hg, Ni, Te, and Zn were detected.

### MICROBIAL COMMUNITIES ASSOCIATED WITH PHYTOREMEDIATION

Microbial communities from the rhizosphere of the arsenic-hyperaccumulating plant *Pteris vittata* and non-rhizosphere samples were examined using GeoChip 3.0 (Xiong et al., 2010). The functional gene diversity was significantly correlated with As concentration. Interestingly, As contaminated rhizosphere samples had higher functional gene diversity than non-rhizosphere samples even though the non-rhizosphere samples had a lower level of As. In addition, greater numbers of As resistance genes, with higher signal intensities, were detected in rhizosphere samples compared to non-rhizosphere samples and very few genes were detected in both environments, suggesting that the rhizosphere and non-rhizosphere microbial communities were distinct. Results suggested that the *P. vittata* rhizosphere may protect the microbial communities from As contamination.

Another study used GeoChip 2.0 to examine microbial communities in Zn- and Cd-contaminated soil microcosms with or without *Thlaspi caerulescens*, a Cd and Zn hyperaccumulator plant (Epelde et al., 2010). Higher numbers of functional genes were detected in the contaminated samples than in uncontaminated samples and in planted samples compared to unplanted. Thirty-five to forty-seven percent of the variation in community structure observed was explained by metal concentrations. All of the Cd and/or Zn resistance genes (12) were detected in the contaminated, planted samples while only 7 were detected in the contaminated/unplanted samples. Substrate-induced respiration, K concentration, and nitrate concentration were the most important environmental variables in determining the functional community structure.

### OIL-CONTAMINATED SITES

The microbial community associated with a bioremediation system comprised of a fluidized bed reactor to clean diesel-contaminated groundwater in Vega Baja, Puerto Rico was examined with the GeoChip 1.0 (Rodríguez-Martínez et al., 2006). Genes involved in the degradation of diesel fuel and other organic contaminants (acetylene, aniline, benzoate, biphenyl, cyclohexanol, methyl tert-butyl ether, naphthalene, phthalate, protocatechuate, and toluene) were detected. Increased signal intensities for genes involved in anaerobic benzoate degradation indicated a shift toward anaerobiosis over time, a conclusion supported by other experimental evidence.

Liang et al. (2009b) examined the effect of different bioremediation treatments on microbial communities using laboratory scale bioremediation systems with sediment from contaminated oil fields and inoculated with oil degrading enrichment cultures. The systems were incubated 242 days, treated with ozone, and incubated an additional 125 days. Many oil degradation genes (benzene, benzoate, catechol, polyaromatic hydrocarbon aromatics, protocatechuate, phthalate) were detected with GeoChip 2.0. Ozonation treatment resulted in an almost 50% reduction in the number of functional genes detected. Gene numbers increased again after a recovery period and the community retained the ability to degrade oil.

Another study used GeoChip 2.0 to characterize microbial communities along an oil contaminant gradient and found a decreased number of functional genes as the contaminant levels increased although genes involved in the degradation of biphenyl, catechol, and protocatechuate increased in the more contaminated samples (Liang et al., 2009a). The most important environmental factors in determining the microbial community structure were oil concentration and soil available nitrogen.

Liang et al. (2011) collected contaminated and uncontaminated soils from five oil fields across China in order to determine whether oil contamination or geographic location played a larger role in determining the microbial community structure. Results from GeoChip 2.0 indicated that communities from uncontaminated sites had higher functional gene diversity than those from contaminated sites in the same geographical area. Overall, the microbial communities clustered based on geographic location; however, when only organic contaminant degradation genes were examined, the contaminated samples clustered together. Geographic location was able to explain ~33% of the microbial community variation observed, oil explained ~10% of the variation, and soil geochemistry explained another 12%, while the remainder (~41%) was unexplained.

GeoChip 4.0 was used to compare microbial communities in oil-contaminated water to those from uncontaminated water in order to understand the effects of the 2010 Gulf of Mexico oil spill (Hazen et al., 2010; Lu et al., 2012a). Results indicated that after only 40 days the presence of the hydrocarbon plume (1100 m depth) caused a significant shift in the microbial community functional structure and composition and that indigenous microorganisms, similar to known petroleum degraders, were stimulated by the hydrocarbon plume. Many genes associated with hydrocarbon degradation were significantly enriched in plume samples (Hazen et al., 2010; Lu et al., 2012a). Genes that were enriched in plume samples included those for naphthalene 1,2-dioxygenase, β-oxidation of benzylsuccinate, cyclohexanone 1,2-monooxygenase, and alkene monooxygenase (Lu et al., 2012a). These findings suggest that the microbial communities in the Gulf of Mexico were capable of intrinsic bioremediation and that the presence of the oil stimulated the oil-degrading community members and were important in determining the fate of the deep-sea oil spill.

### PESTICIDE CONTAMINATION

In a study using GeoChip 2.0 to examine three atrazine-contaminated aquifers and a background site, Liebich et al. (2009) detected more genes in the background site compared to the contaminated sites. The aquifer with the highest level of contamination had the highest number of genes, most involved in contaminant degradation, compared to the other contaminated samples. Atrazine-degradation genes were detected in all contaminated samples and verified by PCR. These results indicated that even small amounts of contaminant were enough to select for specific degrading populations.

River sediments from industrial pollutant and pesticide-contaminated sites were examined with GeoChip 2.0 and the results indicated that contaminant level was not a major driver in these systems (Tacş et al., 2009). Instead, C/N ratio, depth, total Kjeldahl N, and location were the strongest drivers in determining the community structure. Most of the reductive dehalogenation genes detected were from *Dehalococcoides* spp., suggesting that this microorganism may play an important role in contaminant degradation in this system.

### OTHER CONTAMINANTS

GeoChip 2.0 was used to examine phenanthrene-spiked soil microcosms to examine the effect of phenanthrene on microbial communities (Ding et al., 2012). Communities were examined after a 21-day incubation and compared with communities from day 0. A larger number of genes were detected in spiked soils compared to the control soils. Genes showing an increase in the spiked soils included dioxygenases involved in aromatic compound degradation, genes involved in the degradation of PAHs (*nahA*, *rhda*, *nahQ*, *narR*), and genes involved in the degradation of one-ring aromatic compounds. In addition, an overall shift in community composition and structure was noted in spiked soils as determined by non-metric multidimensional scaling.

Another study examined microbial communities associated with a leachate-contaminated landfill using GeoChip 3.0 (Lu et al., 2012b). Groundwater samples were collected from wells along a flowpath of the landfill. Communities directly under the landfill and in the closest well had significantly lower functional gene diversity and richness. Genes involved in the anaerobic degradation of organic contaminates such as aromatic acids (*bclA*, *bbs*, *tutFDG*), phenoxyacetic acid herbicides (*ftdA*) atrazine (*atzABC*, *trzN*, *trzA*, *trzE*) were detected in all wells. Based on canonical correspondence analysis, the environmental variables (pH, sulfate, ammonia, and dissolved organic carbon) had significant effects on the community structure.

## SUMMARY

The GeoChip arrays have been shown to be powerful tools in linking microbial function to ecosystem processes and are able to provide sensitive, specific, and potentially quantitative information. Use of this array in bioremediation studies have expanded our understanding of the microbial processes and communities at work in these sites and provide information necessary for the successful improvement and application of bioremediation strategies. Over the past decade, great improvements have been made in regards to microarray technology, design, and application. However, there are still technical hurdles that need to be overcome to further improve sensitivity and specificity in addition to better methods of nucleic acid extraction and purification. Improved bioinformatics tools are also needed to assist with data processing and analysis.

## Conflict of Interest Statement

The authors declare that the research was conducted in the absence of any commercial or financial relationships that could be construed as a potential conflict of interest.

## Footnotes

1 http://hmmer.wustl.edu/

2 http://ieg.ou.edu/

3 ieg.ou.edu/microarray/
